# Role of Cbl-PI3K Interaction during Skeletal Remodeling in a Murine Model of Bone Repair

**DOI:** 10.1371/journal.pone.0138194

**Published:** 2015-09-22

**Authors:** Vanessa Scanlon, Do Yu Soung, Naga Suresh Adapala, Elise Morgan, Marc F. Hansen, Hicham Drissi, Archana Sanjay

**Affiliations:** 1 Department of Orthopaedic Surgery, University of Connecticut Health Center, Farmington, CT, United States of America; 2 Department of Mechanical Engineering, Boston University, Boston, MA, United States of America; 3 Center for Molecular Medicine, University of Connecticut Health Center, Farmington, CT, United States of America; 4 Department of Genetics and Genome Sciences, University of Connecticut Health Center, Farmington, CT, United States of America; University of Oulu, FINLAND

## Abstract

Mice in which Cbl is unable to bind PI3K (YF mice) display increased bone volume due to enhanced bone formation and repressed bone resorption during normal bone homeostasis. We investigated the effects of disrupted Cbl-PI3K interaction on fracture healing to determine whether this interaction has an effect on bone repair. Mid-diaphyseal femoral fractures induced in wild type (WT) and YF mice were temporally evaluated via micro-computed tomography scans, biomechanical testing, histological and histomorphometric analyses. Imaging analyses revealed no change in soft callus formation, increased bony callus formation, and delayed callus remodeling in YF mice compared to WT mice. Histomorphometric analyses showed significantly increased osteoblast surface per bone surface and osteoclast numbers in the calluses of YF fractured mice, as well as increased incorporation of dynamic bone labels. Furthermore, using laser capture micro-dissection of the fracture callus we found that cells lacking Cbl-PI3K interaction have higher expression of Osterix, TRAP, and Cathepsin K. We also found increased expression of genes involved in propagating PI3K signaling in cells isolated from the YF fracture callus, suggesting that the lack of Cbl-PI3K interaction perhaps results in enhanced PI3K signaling, leading to increased bone formation, but delayed remodeling in the healing femora.

## Introduction

The E3 ubiquitin ligase Cbl is a multi-domain adaptor protein [1], which regulates bone resorption by interacting with several molecules in osteoclasts[2–6]. Cbl was also shown to control ubiquitylation of signaling molecules and regulate proliferation, differentiation, and survival of human mesenchymal-derived osteoblasts [7]. Cbl’s expression is decreased in primary bone tumors, and ectopic Cbl expression reduces bone tumorigenesis by promoting tyrosine kinase receptor degradation [8]. Thus, Cbl’s role in fine tuning signaling pathways in bone turnover appears to be important in both normal and pathological conditions.

Phosphatidylinositol-3 Kinase (PI3K), a lipid kinase, plays a central role in regulating cell proliferation, survival, and migration [9]. The PI3K enzyme is a heterodimer of p110 catalytic and p85 regulatory subunits. The primary function of the p85 subunit is to bind and stabilize the p110 subunit [10]. PI3K signaling plays an important role in skeletal homeostasis [11, 12]. Pan-specific PI3K inhibitors inhibit osteoblast growth and survival induced by a wide range of extracellular ligands [13–16] suggesting that PI3K signaling positively regulates the number of available osteoblast precursors. PI3K/AKT signaling pathway in concert with BMP-2 signaling mediates osteoblast differentiation [17, 18]. A reduction in alkaline phosphatase (ALP) activity and osteocalcin mRNA expression was reported in p85α^-/-^ deficient mesenchymal stem cells suggesting a role for PI3K signaling in osteoblast differentiation [19]. The phosphatase and tensin homolog (PTEN), is a direct antagonist of PI3K. Deletion of PTEN in mature OBs led to increased bone mass [20] and its loss in osteoprogenitors resulted in increased proliferation [21]. Therefore, regulation of PI3K plays a significant role during osteogenesis.

Cbl is a major protein that interacts with PI3K and regulates its activity. Phosphorylation of tyrosine Y737 in the YEAM motif on Cbl is required for the binding of the SH2 domain of the p85 subunit of PI3K [22–24]. The substitution of tyrosine 737 to phenylalanine abrogates Cbl’s interaction with PI3K [23, 25] and decreases osteoclast function [5].

To study the impact of Cbl-PI3K interaction, knock-in mice in which the tyrosine 737 was substituted with phenylalanine (YF mice) were used [26]. Our initial characterization of YF mice during normal bone homeostasis revealed decreased bone resorption [27–29] and enhanced bone formation resulting in increased bone volume [30]. Moreover, mechanical testing of intact femora from these mice revealed a significant increase in peak moment and yield displacement, indicating that while there is more bone present, impaired remodeling may be contributing to pathological bone quality [30]. Recently, we have shown that YF mice do not undergo significant bone loss following ovariectomy [31]. Together, these data indicate that increase in bone volume in YF mice is due in part, to enhanced osteogenesis and increased osteoblastic function *in vivo*. Despite these changes in bone remodeling in adult mice, changes in skeletogenesis during development in YF mice are transient, making it a challenge to study the role of Cbl-PI3K interaction in developing long bones.

Bone is a dynamic organ capable of regenerating in response to injury. To better elucidate the role of Cbl-PI3K interaction during bone formation and remodeling of long bones, we utilized a mid-diaphyseal femoral fracture model, which heals through both intramembranous and endochondral ossification [32]. The repair of fractured long bones consists of chondrogenic and osteoblastic phases of callus formation followed by osteoclast-mediated remodeling. The regulation of intracellular signaling pathways within these cell types plays an important role in their function, however the molecules and mechanisms responsible for this regulation remain largely unknown.

Here, we report a novel role for Cbl-PI3K interaction in bone repair. Our results show that abrogation of Cbl-PI3K interaction perturbs bone regeneration, affecting both osteoclasts and osteoblasts during the healing process.

## Materials and Methods

### Experimental Animals and Surgical Procedure

Knock-in mice with a point mutation on the PI3K binding site of Cbl (CblY737F; CblYF/YF, henceforth referred to as YF mice) were previously described [26]. Mice were maintained on a C57BL/6x129SvJ background and animal procedures were conducted according to protocols approved by the University of Connecticut Health Center Animal Care Committee. We determined that using young adult female mice between 7 and 9 weeks of age would be appropriate to study fracture healing in the mixed background based on previous findings [33]. They were given 0.1 mg/kg Buprenorphine by subcutaneous injection just prior to the procedure, and twice a day for three days following the fracture. Animals were anesthetized with 4% Isoflurane and mid-diaphyseal femoral fractures were performed on left limbs as described previously [34–36] using a three-point bending device [37]. Mice were euthanized on days 7, 14, 21, 28 and 35 days post fracture by CO2 narcosis followed by cervical dislocation.

### Imaging analyses

Fractures were radiographed using a Faxitron Cabinet X-Ray System (Faxitron X-Ray Corporation, Lincolnshire, IL) set at 26kV for 6 seconds under anesthesia on days 0, 7, 14, 21, 28 and 35 post-fracture to monitor initial pin fixation and ongoing callus formation and resorption. Volumetric analyses to determine total callus volume and bony callus volume were conducted at the MicroCT Imaging Facility at the University of Connecticut Health Center using cone-beam micro-focus X-ray computed tomography (Scanco Medical AG μCT40, Bruttisellen, Switzerland). Within the callus, newly formed bone was defined by excluding original cortical bone and contouring the edge of the callus on each 12μm 2D slice. Serial tomographic images were acquired and three-dimensional 16-bit gray scale images were reconstructed as previously described [38].

### Histology and Histomorphometry

Histology and histomorphometry were performed as previously described. Briefly, bones were fixed in 10% neutral buffered formalin for 7 days, decalcified in 14% EDTA (pH 7.2) for 14 days and embedded in paraffin. 5μm sections were cut and stained with Safranin O and Fast Green. Some sections were also stained for TRAP activity using a commercial kit (Kamiya Biomedical Company LLC, Seattle, WA). Sections were viewed on a Nikon Eclipse 50i microscope; images were captured using a Q Imaging Retiga 2000R camera; processing was done using NIS Elements software. Histomorphometry was performed using Osteomeasure to measure Total Callus Area (TA), Bony Callus Area (BA), Cartilaginous Callus area (CA), number of TRAP^+^ cells, Osteoclast surface, Erosion surface, and Bone Surface, Osteoblast Surface/ Bone Surface (Ob.S./B.S.), ObS was defined as three or more cuboidal shaped cells in a row directly adjacent to bone [39]. For some experiments, fractured femora were fixed in 10% buffered formalin for seven days, then 30% sucrose overnight at 4°C prior to embedding in OCT media. 5 μm frozen sections were cut and stained with Calcein blue to visualize mineralized tissue.

### Dynamic Bone Labeling

Mice were given intraperitoneal injections of Alizarin Complexone (30mg/kg) (Sigma-Aldrich, St. Louis, MO) four days prior to sacrifice, and Calcein (10mg/kg) (Sigma-Aldrich, St. Louis, MO) one day prior to sacrifice. At the time of harvest, fractured femora were fixed in 10% buffered formalin for seven days, then 30% sucrose overnight at 4°C prior to embedding in OCT media. 5 μm frozen sections were cut and mounted on glass slides for imaging. Fluorescent images were taken on a Leica (microscope info).

### Laser Capture Microdissection and real time qPCR

Deparaffinized sections were rehydrated, and lightly stained with H&E. Laser capture microdissection (LCM) was performed using the Arcturus PixCell II system (Arcturus, Engineering, Mountain View, CA) using an infrared laser of 20μm diameter, 100mW power, and 2.5ms pulse width to capture all cells within the callus. Energy from the laser bonded the cells to a thin film polymer substrate on the LCM caps (Arcturus Engineering), which were then lifted free from the tissue section and transferred to a microcentrifuge tube. To extract RNA, captured cells were lysed in a buffer containing 0.5% NP-40 and RNAsin Plus (Promega, Fitchburg, WI). cDNA was prepared using ABI High Capacity RT kit (Life Technologies, Grand Island, NY). Linear amplification of target cDNA was performed with Takara Taq (Clonetech Laboratories Inc., Mountain View, CA). qRT-PCR was performed with the amplified cDNA using SYBR Green Master Mix (Life Technologies, Grand Island, NY) and validated custom designed primers (Table 1) on ABI Step One Plus Real Time PCR System using the following program: 95°C for 10 minutes, 40 cycles of 95°C for 15 seconds and 57°C for 1 minute. Product specificity was confirmed by a melting curve, and relative expression was determined using delta-delta CT analysis normalizing to β actin expression.

**Table 1:** LCM Primer Sequences.

Target Name	Forward Primer (5’-3’)	Reverse Primer (5’-3’)	Product Size
Runx2	CCACCACTCACTACCACACG	CACTCTGGCTTTGGGAAGAG	63bp
Osterix	GATGGCGTCCTCTCTGCTTG	GCCATAGTGAGCTTCTTCCTCAA	40bp
TRAP	GCAGCTCCCTAGAAGATGGATT	ATTTGTAGGCCCAGCAGCAC	50bp
Calcitonin Receptor	AGGGGAATTGTCCTCAACACC	CGGCTGGCTCTCCTGACTTG	40bp
Akt2	GGGAGACCCAAGACGATACTG	TTCACACGCTGTCACCTAGC	87bp
IKKα	GCCCCCTACATTAGCAGACC	TGTGCTAACGTCTCTCACACA	58bp
Rac1	CAGATGCAGGCCATCAAGTGT	TTACCAACAGCTCCGTCTCC	50bp
Rac2	TCAGATGCAATGCAGGCCA	CACGGCTCCATCACCCAC	51bp

### Mechanical Testing

Fractured femora and contralateral intact femora were collected 21 and 35 days post-fracture and mechanical testing was performed as previously described [40]. The proximal and distal ends of each femur were embedded in polymethyl methacrylate (Bosworth, Skokie, IL) in 1 cm square aluminum tube casings. The longitudinal axis of the bone was centered in the casing so as to be collinear with the axis of torsion. The gage length was defined as the length of the specimen that spanned the distance between the two casings and was calculated as the average of four measurements taken at 90° increments around the specimen circumference. Femora were then secured in the test frame (MT55, Instron, Norwood, MA). The distal ends of the specimens were rotated inward at a rate of 1 degree per second. Torque was measured with a 2.25-N·m transducer and angular displacement with an optical encoder. Torsional strength was defined as the maximum torque sustained by the specimen. Work was calculated as the area under the torque-angular displacement curve up to the peak torque.

### Statistical analyses

Results were analyzed using Student's *t-*test. Data are presented as means ± SD. *p*-values < 0.05 were considered statistically significant.

## Results

### Loss of Cbl-PI3K interaction results in larger callus formation during fracture healing

**Fig 1:**
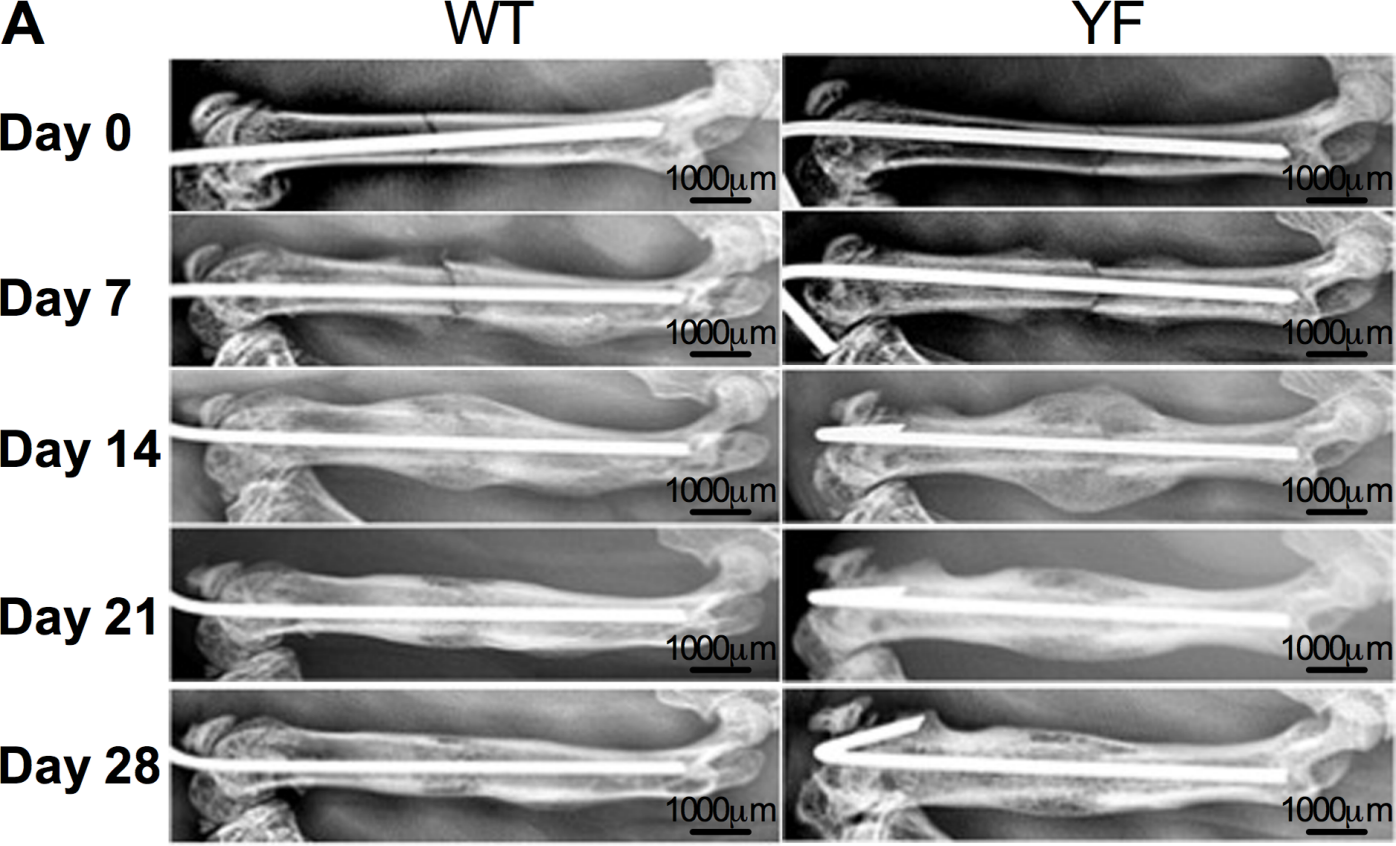
Radiographic images show increased callus formation and delayed callus resorption in mice lacking Cbl-PI3K interaction. Representative WT and YF mice were X-rayed once a week for four weeks to observe pin fixation, confirm fracture, and monitor callus formation.

Following the discovery that, in mice, a single point mutation that disrupts the interaction between Cbl and the p85 subunit of PI3K is capable of uncoupling bone resorption and bone formation during skeletal homeostasis [27, 29, 30], we examined whether it influenced skeletal remodeling during femoral fracture repair. Radiographic evaluation of the mid-diaphyseal fractures over a course of 28 days showed callus formation in fractured bones in both genotypes. By day 14, a much larger callus with more radiolucency was visible in YF fractured femora. As resorption of the callus progressed, by day 28, YF femora had more radiolucent fracture callus compared to WT. While both WT and YF mice had bony union, the latter also had more residual callus at later time points suggesting a delay in the healing process (Fig 1). Radiographic scoring of fractures over the course of healing also revealed enhanced early fracture callus formation in YF mice compared to WT (S1 Fig).

**Fig 2:**
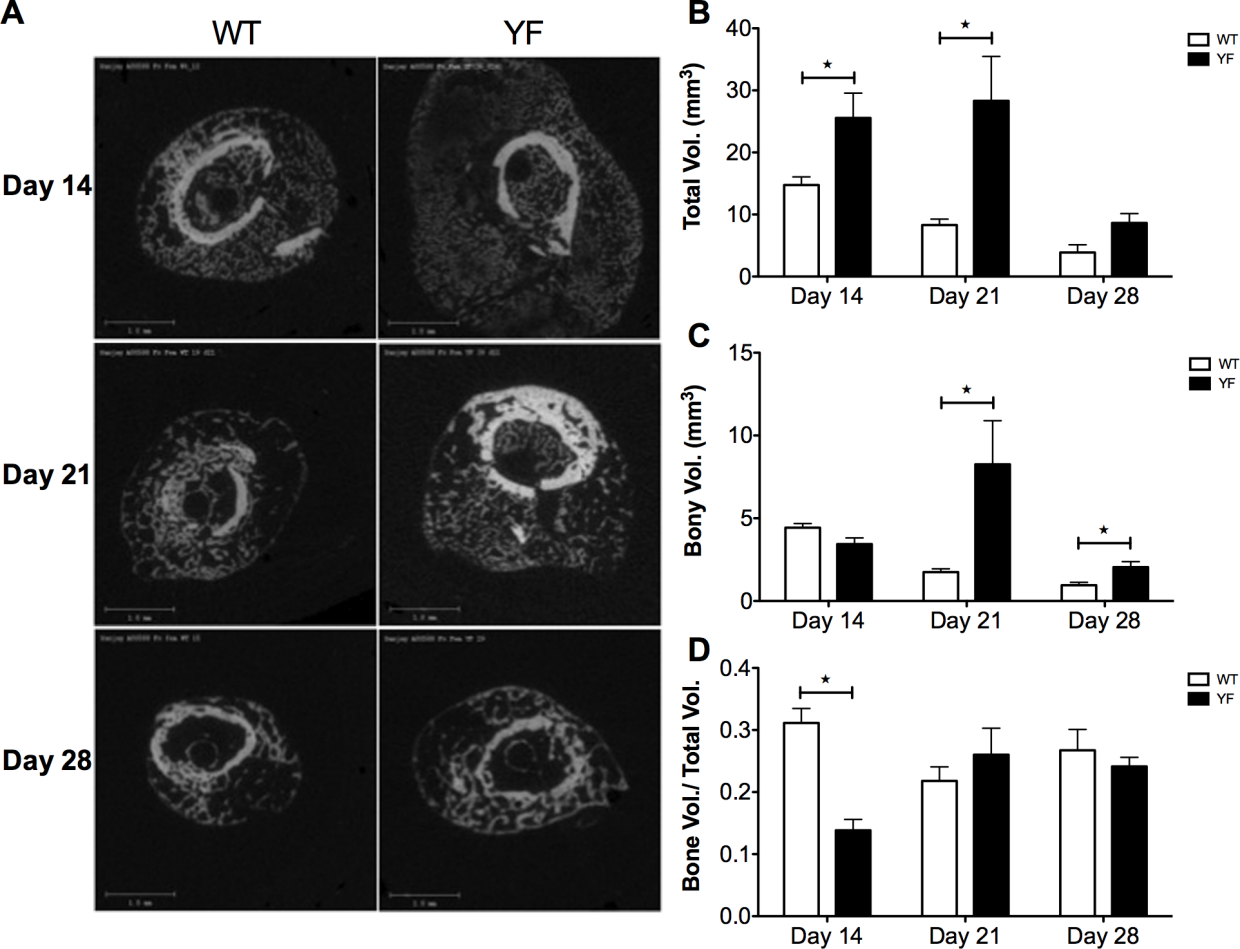
MicroCT measurements showed increased total callus and bony volume in mice lacking Cbl-PI3K interaction. A. Fractured femora were harvested from WT and YF mice at 14, 21 and 28 days post-fracture, and scanned by MicroCT. Bar charts show B. Total callus volume C. Bony callus volume D. Ratio of bony over total callus volume. n = 5–6, *p<0.05 vs. WT.

MicroCT imaging showed increased callus size with a mineralized void at the center of calluses in the YF mice compared to WT mice at 14 days post-fracture (Fig 2A). In fractured WT femora, total callus volume was greatest at 14 days post-fracture, and then continuously decreased throughout the duration of the study. Quantitative volumetric analysis confirmed that the total callus size was significantly larger in the YF mice at day 14 and day 21 compared to WT (Fig 2B and S2 Fig). Similarly, compared to WT, bony volume of YF calluses remained increased during remodeling at days 21 and 28 (Fig 2C and S2 Fig). We also determined the ratio of bony callus volume to total callus volume. Since the total callus volume was much larger in YF mice at day 14 without having a significant difference in bony callus volume, the ratio of bony callus volume to total callus volume was significantly lower in YF mice as compared to WT mice. However, at 21 and 28 days post-fracture the ratio was comparable between the genotypes (Fig 2D). Together, these results indicate that the lack of Cbl-PI3K interaction is concomitant with enhanced bone formation in response to fracture repair.

### Histological assessment showed delayed remodeling in YF calluses

**Fig 3:**
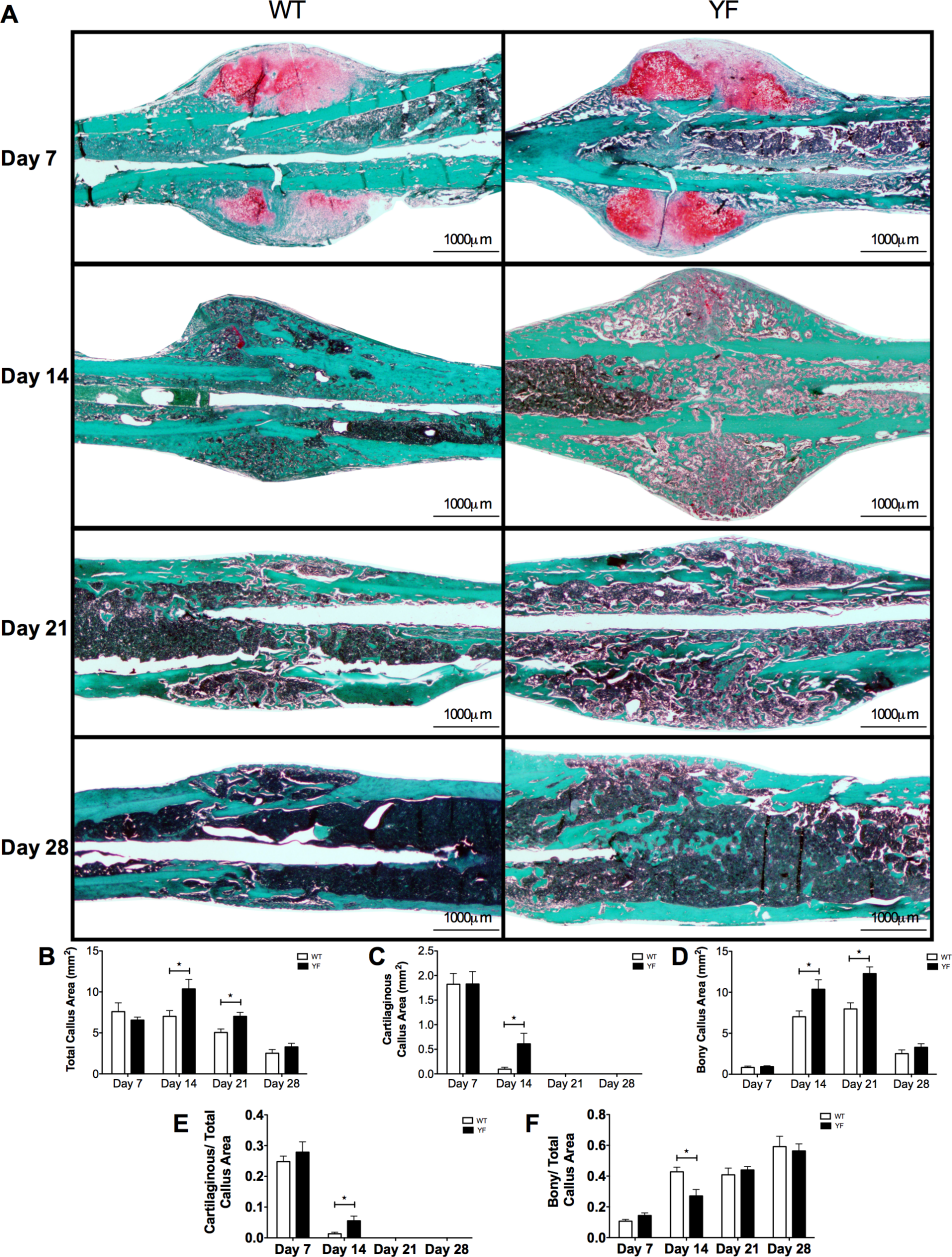
Increased cartilaginous and bony matrix in remodeling fracture calluses of YF mice. For histological analysis, mid-sagittal sections of fractured femora were stained with Safranin O and fast green and quantitated by static histomorphometry. **A.** Representative sections at 7, 14, 21, and 28 days post-fracture. Bar charts show **B.** Total callus area **C.** Cartilaginous matrix area **D.** Bony matrix area. **E.** Ratio of cartilaginous matrix area over total callus area **F.** Ratio of bony matrix area over total callus area. n = 5–6, *p<0.05 vs. WT.

The imaging analyses were complemented with histological and histomorphometric analyses of Safranin-O/Fast green-stained sections of fracture calluses of WT and YF mice at days 7, 14, 21 and 28 post-fracture (Fig 3A). Total callus size was 48 and 40% larger (p<0.05) in YF mice at days 14 and 21 post-fracture respectively as compared to WT (Fig 3B). There were no significant differences in total callus size, bony callus, or cartilaginous callus area measured by histomorphometry at 7 days post-fracture. At 14 days post-fracture, while the majority of cartilaginous callus in WT fractured bone was replaced with bone, about 5.5-fold more (p<0.05) residual cartilage remained in calluses from YF mice (Fig 3C), even when normalized to the total callus area (Fig 3E). Histomorphometric measurements showed that at 14 days post-fracture not only the cartilage content, but also the bony callus content was 48% increased (p<0.05) in YF mice compared to WT (Fig 3D). However, when normalized to the total callus area, the ratio of bony to total callus area was 37% reduced (p<0.05) in YF samples (Fig 3F). Furthermore, compared to WT samples, increased bony callus area persisted in YF mice 21 days post-fracture (Fig 3D), but this difference was not significant when normalized to total callus area (Fig 3F). Together these results indicated that the lack of Cbl-PI3K interaction did not alter soft callus formation, but resulted in increased bony callus formation, and slower remodeling probably due to increased bone formation and delayed bone resorption.

### YF remodeling calluses were less resistant to torsional force

**Fig 4:**
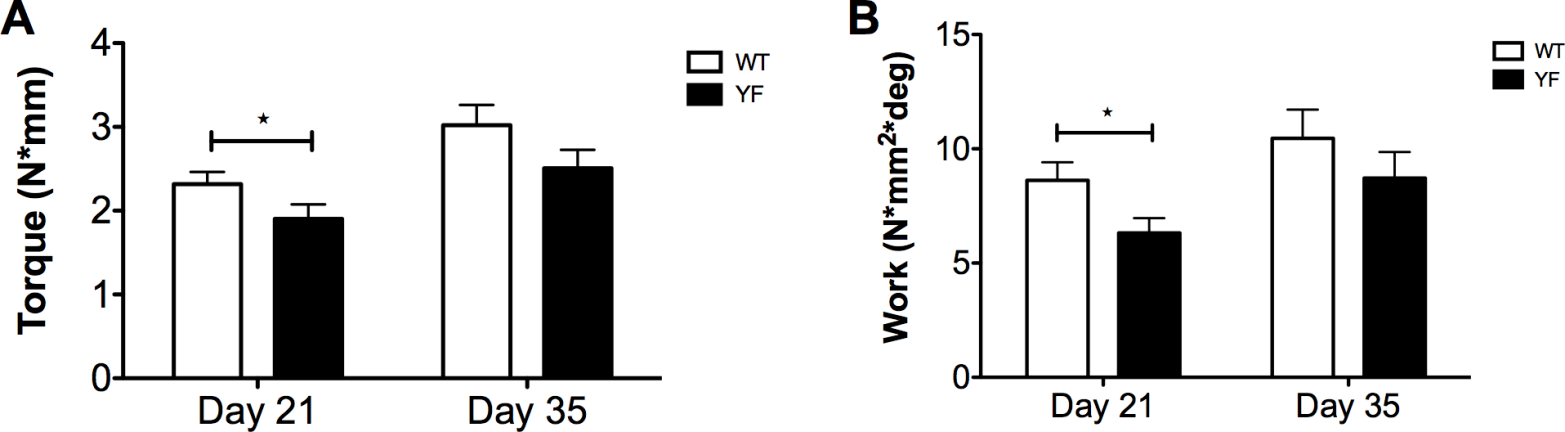
Fractured femora lacking Cbl-PI3K interaction are delayed in restoring mechanical strength. Torsion testing was performed on fractured femora during remodeling of the hard callus at 21 and 35 days post-fracture. Bar graphs show **A.** Peak torque and **B.** Work. n = 11 *p<0.05 vs. WT.

Given that the YF fracture calluses had increased callus size and bone volume during the remodeling and resorption phases of fracture healing (Figs 2 and 3), we next performed torsional tests of remodeled calluses to determine the torque and energy required to break the newly formed bone. Mechanical testing was performed on isolated fractured femora of WT and YF mice 21 and 35 days post-fracture. The results show that at day 21, YF fractured femora required 20% less (p<0.05) torque (Fig 4A), and 27% less (p<0.05) work to cause failure compared to WT (Fig 4B). In contrast, maximum torque and work resolved to no significant difference between the two genotypes at day 35 post-fracture (Fig 4). These data suggest that, despite increased bone volume at 21 days post-fracture, YF fractured femora are weaker, probably due to a delay in the remodeling of the mineralized bony callus (as seen by microCT and histology) resulting in pathological bone, but by 35 days post-fracture, remodeling progresses in both genotypes, and the strength of the bones is comparable.

### Abrogation of Cbl-PI3K interaction resulted in increased osteoblast surface/bone surface, callus mineralization, and Osterix expression in bony calluses

**Fig 5:**
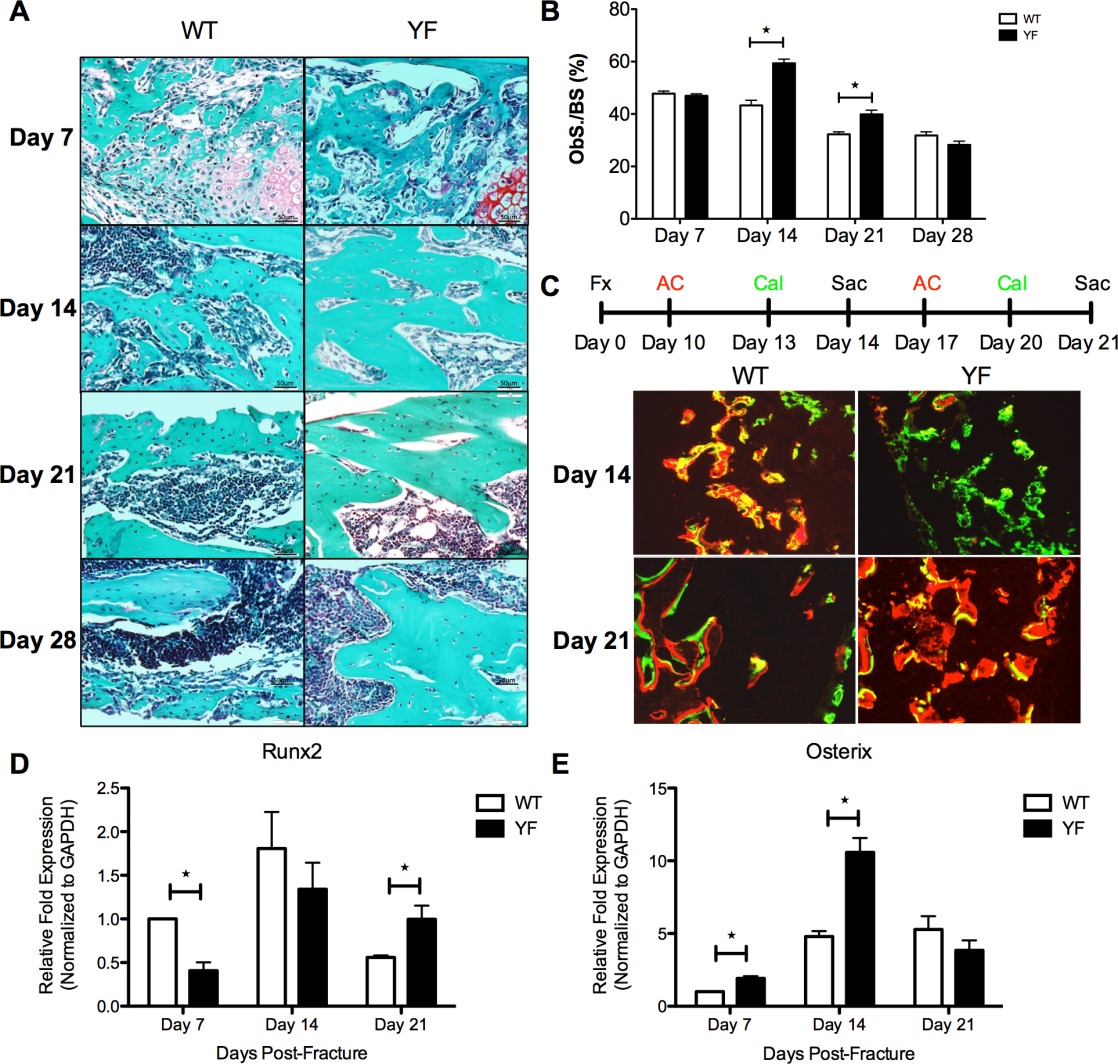
Lack of Cbl-PI3K interaction results in increased osteoblast surface, incorporation of dynamic bone labels, and up-regulation of master transcription factor Osterix in remodeling fracture calluses. Mid-saggital sections of fractured femora were stained with Safranin O and Fast Green, and counterstained with Hematoxylin. **A.** Representative sections at 7, 14, 21, and 28 days post-fracture showing bony regions of fracture calluses. **B.** Osteoblast surface over bone surface (ObS/BS) measured by static histomorphometry. n = 6, *p<0.05 vs. WT. **C.** Mice were injected with Alizarin Complexone (red) 4 days prior to sacrifice, and Calcein (green) 1 day prior to sacrifice. Mid-sagittal sections of fractured femora were used to image incorporation of dynamic bone labels in the center of the fracture calluses at 14 and 21 days post-fracture. Cells from sections of calluses at 7, 14, and 21 days post-fracture were harvested by LCM, and expression of **D.** Runx2, and **E.** Osterix were quantified by qRT-PCR n = 6, *p<0.05 vs. WT.

To determine, if, the observed increases in the bony callus surfaces in the YF mice could be due to enhanced bone formation, we next evaluated bone formation parameters by histological and histomorphometric analyses. Histomorphometric analysis of OB surface over bone surface (ObS/BS) on Fast Green stained sections counterstained with Hematoxylin (Fig 5A) revealed a 37% (p<0.05) and a 25% (p<0.05) increase in YF during remodeling at days 14 and 21 post-fracture, respectively (Fig 5B). We attempted to obtain quantitative measurements of bone formation within the callus by dynamic bone labeling with Alizarin Complexone (AC) and Calcein. At 10 days post-fracture, YF samples incorporated much less AC into the center of the callus compared to WT. However, at 13 days post-fracture, there did not appear to be a substantial difference in Calcein incorporation in the center of the callus of YF compared to WT mice (Fig 5C). Furthermore, there was more AC incorporation at the center of YF fracture calluses 17 days post-fracture, and Calcein incorporation 20 days post-fracture as compared to the WT samples (Fig 5C). Given the radial nature of bone deposition in the fracture callus, measuring the distance between labels is difficult and would be inaccurate. However, on frozen sections using a Calcein Blue stain to identify mineralized bony tissue [41–43], we observed visually more stain in YF sections at days 14 and 21 post-fracture (S3 Fig), which further supported the finding that YF mice form more bone in response to fracture. We confirmed the increase in bone matrix production in the YF fracture calluses by examining the expression of Runx2 [44] and Osterix [45], two master regulators of the OB lineage [46]. Laser Capture Microdissection of the whole callus followed by qRT PCR analysis showed a 2-fold (p<0.05) reduction in Runx2 expression at 7 days post-fracture in YF samples compared to WT, no change at 14 days post-fracture, and a 2-fold increase (p<0.05) at 21 days post-fracture (Fig 5D). Conversely, we quantitated 2-fold and 1.67-fold increases (p<0.05) in Osterix expression at both 7 and 14 days post-fracture, respectively, in YF samples compared to WT. Together, these results support our underlying hypothesis that lack of Cbl-PI3K interaction results in increased bone formation in response to bone fracture.

### Lack of Cbl-PI3K interaction resulted in increased numbers of osteoclasts despite diminished resorptive capacity and delayed remodeling

**Fig 6:**
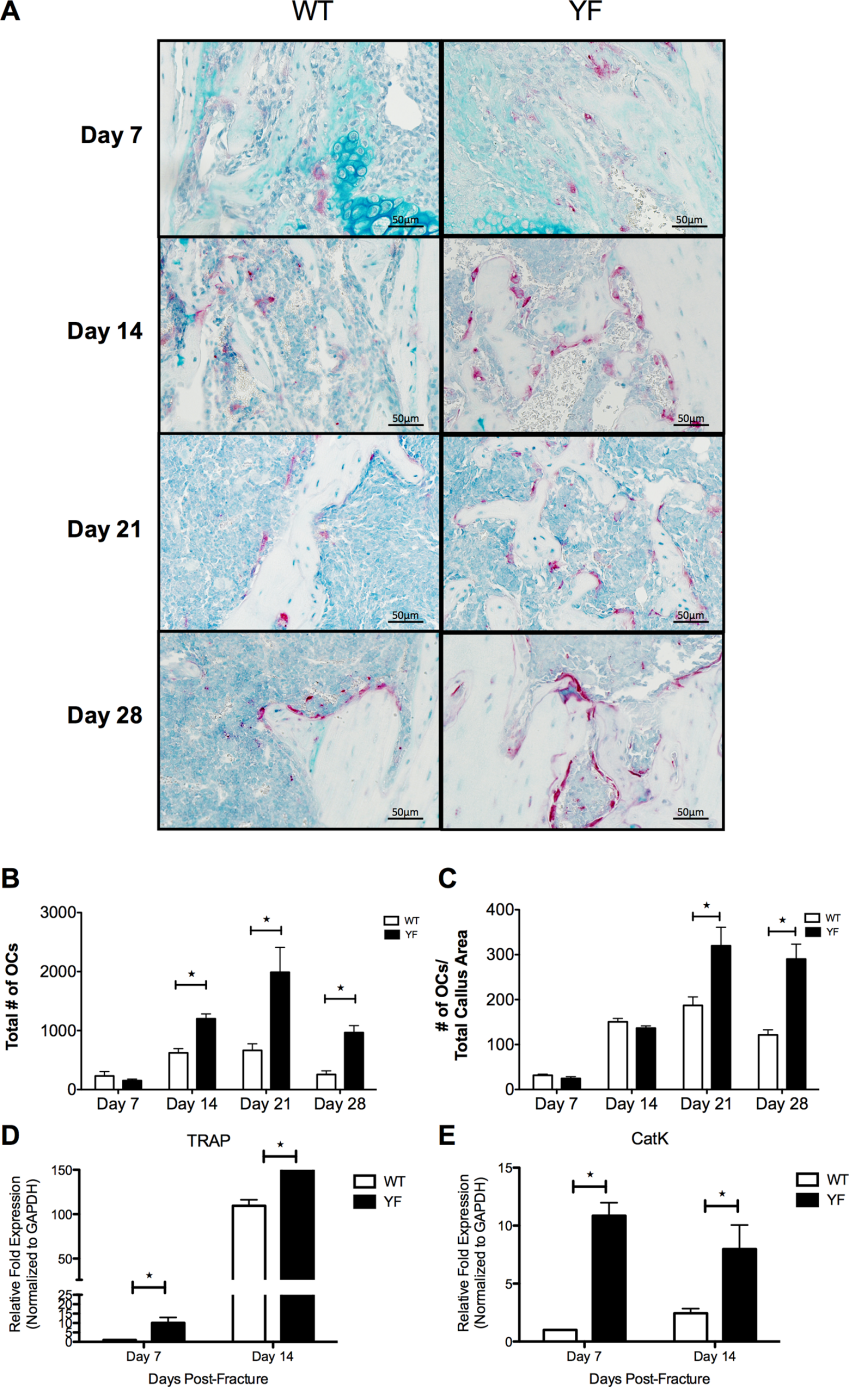
Lack of Cbl-PI3K interaction results in increased osteoclast number, and up-regulation of osteoclastic markers TRAP and Cathepsin K in remodeling fracture calluses. Mid-sagittal sections of fractured femora were TRAP stained and counterstained with Alcian Blue and Hematoxylin. **A.** Representative sections at 7, 14, 21, and 28 days post-fracture. Bar charts show **B.** Total number of osteoclasts and **C.** Ratio of osteoclast number over total callus area. n = 5–6, *p<0.05 vs. WT. Cells from sections of calluses at 7 and 14 days post-fracture were harvested by LCM, and expression of **D.** TRAP and **E.** Cathepsin K were quantified by RT-PCR. n = 3, *p<0.05 vs. WT.

To address the possibility that the increases observed in the residual cartilaginous and bony calluses of YF mice could be due to delayed remodeling and bone resorption, we examined the number of osteoclasts (OCs) at days 7, 14, 21, and 28 post-fracture on TRAP stained sections (Fig 6A). No significant difference between the numbers of OC was observed at 7 days post-fracture in the WT and YF fracture calluses. However, by day 14 there was a 2-fold increase (p<0.05) in OC numbers in YF calluses compared to WT calluses, which persisted at days 21 and 28 post-fracture (Fig 6B). The increased OC number in YF fracture calluses remained even when normalized to the total callus area (Fig 6C). Laser Capture Microdissection of the whole callus followed by RT PCR analysis showed a 10-fold, and 1.8-fold (p<0.05) increase in TRAP expression at days 7 and 14 post-fracture in YF samples, respectively compared to WT (Fig 6E). Similarly, Cathepsin K expression was also 10.8-fold and 2.3-fold up-regulated (p<0.05) in YF calluses at days 7 and 14 post-fracture, respectively. Despite the appearance of large, polarized osteoclasts directly adjacent to bony callus (S4 Fig), osteoclasts within the fracture callus had diminished resorptive activity as evidenced by a significant decrease in erosion surface (Table 2). This observation is in agreement with our previous reports in YF mice demonstrating decreased osteoclast-mediated bone resorption both *in vivo* and *in vitro* [27–29]. These data suggest that the lack of Cbl-PI3K interaction results in increased osteoclast numbers in the remodeling fracture callus, even though remodeling of the callus matrix is slower compared to WT.

**Table 2:** Histomorphometric parameters of bone resorption.

	WT day 14	YF day 14	WT day 14	YF day 14
Osteoclast Surface (mm)	1.600 + 0.20	3.600 +0.20 *	2.033 ± 0.251	3.833 +0.251*
Erosion Surface (mm)	0.900 + 0.10	0.950 + 0.050	1.167 ± 0.152	1.133 +0.251
Bone Surface (mm)	10.00 + 1.00	8.400 +0.529	11.00 ±1.000	9.867 +1.026
Erosion Surface/Osteoclast Surface	0.563 +0.007	0.264 +0.001*	0.573 ± 0.0232	0.330 +0.106*
Erosion Surface/Bone Surface	0.091 +0.019	0.113 +0.002	0.105 ± 0.004	0.114 +0.013
Osteoclast Surface/Bone Surface	0.162 +0.036	0.429 +0.008*	0.187 ± 0.0062	0.353 +0.0145*

Mid-sagittal sections of fractured femora were TRAP stained and counterstained with Alcian Blue and Hematoxylin. Three sections/mouse and 3 mice/genotype at 14 and 21, days post-fracture were analyzed. Osteoclast Surface, erosion surface and bone surface as determined by histomorphometry are shown. Values shown are mean ± SD from WT mice n = 3, YF mice n = 3

* p<0.05 was considered statistically significant as compared to respective controls using analysis of variance with post-hoc analysis (ANOVA) with Bonferroni post-hoc test.

### Lack of Cbl-PI3K interaction may result in increased PI3K signaling in cells contributing to the fracture callus

**Fig 7:**
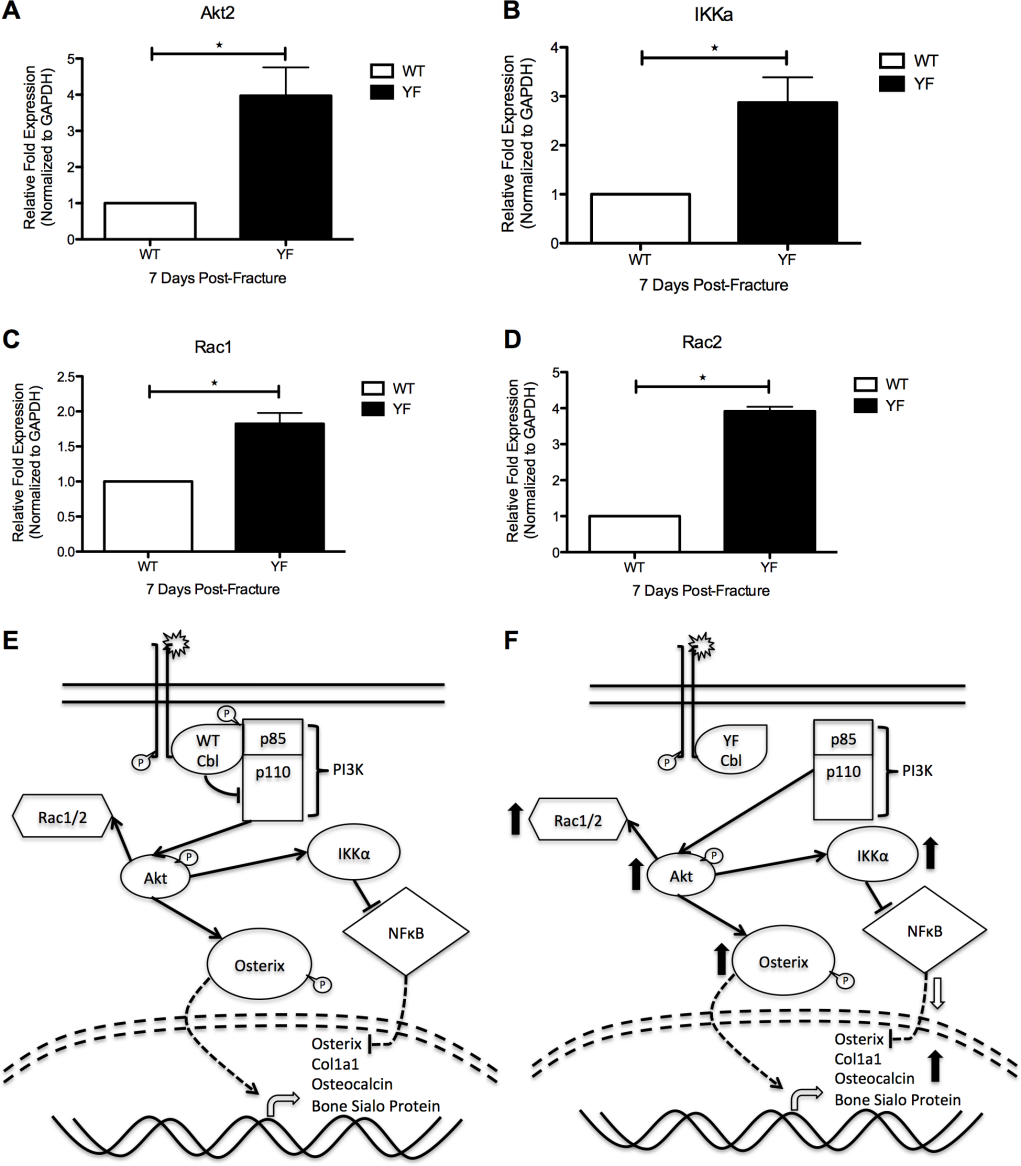
Molecular and functional changes in osteoblasts and osteoclasts of YF fracture calluses may be due to increased PI3K signaling. Cells from sections of fracture calluses at 7 days post-fracture were harvested by LCM and expression of **A.** Akt2 **B.** Ikkα **C.** Rac1, and **D.** Rac2 were quantified by RT-PCR. n = 3 *p<0.05 vs. WT. **E.** Proposed schematic of molecular mechanism in WT mice. **F.** Proposed schematic of molecular mechanism in the absence of Cbl-PI3K interaction (YF mice).

Previously, we reported that disrupted Cbl-PI3K interaction in the YF mutants resulted in increased OC number yet decreased resorptive capacity. Moreover, increased OB number with increased bone formation capacity was also observed in adult mice lacking Cbl-PI3K interaction under bone homeostasis and dynamic bone remodeling conditions [27, 29, 30]. These changes were attributed to increased PI3K signaling, subsequent to the lack of PI3K sequestration by Cbl [47]. The data above point to similar cellular events in the fracture calluses as previously seen during skeletal homeostasis in these mice. Thus, we postulated that enhanced bone formation and delayed remodeling of the callus during fracture healing may be attributed to changes in PI3K signaling. Therefore, we isolated cells from the fracture calluses of WT and YF mice by LCM, and examined the expression of genes known to be involved downstream of PI3K in the signaling cascade. Expression of Akt2 was up-regulated in the YF fracture calluses by approximately 4-fold (p<0.05) over WT samples (Fig 7A). Similarly, expression of IKKα was 3-fold (p<0.05) higher in YF samples compared to WT (Fig 7B). Moreover, Rac1 and Rac2, which have been shown to be positively regulated by PI3K signaling [48] were also up-regulated by 2-fold, and 4-fold (p<0.05) respectively in cells isolated from YF fracture calluses compared to those of WT (Fig 7C and 7D). Taken together, this gene expression pattern suggests that PI3K signaling is increased in cells lacking Cbl-PI3K interaction, as previously seen in these mice under bone homeostasis and dynamic bone remodeling conditions.

## Discussion

Here, we report a novel role for PI3K signaling during bone repair. The key observations of this study were that the absence of Cbl-mediated sequestration of PI3K resulted in a net increase in the total callus size, and the amount of newly formed bone in response to fracture, without any apparent effect on soft callus formation (Figs 2 and 3). At the same time, the lack of Cbl-PI3K interaction also resulted in delayed remodeling of the soft callus to bony callus (Fig 3), as well as remodeling and resorption of the bony callus, despite increased OC numbers (Fig 6), which resulted in delayed restoration of strength in the fractured femur (Fig 5).

The fact that the gross morphology of the soft callus was similar between the YF and WT, and no changes were noted between the two genotypes in the expression of hallmarks of cartilaginous tissue in the calluses markers (data not shown), suggests that the Cbl-PI3K interaction may not play a significant role in this early stage of fracture healing. The observed increase in the overall size of the bony callus in animals lacking Cbl-PI3K interaction combined with increased ObS/BS, enhanced deposition of dynamic bone labels, and increased Osterix expression in later stages of healing suggest that Cbl-mediated regulation of PI3K signaling plays an important role in bone regeneration. It is known that both Runx2 [44, 46] and SP7/Osterix [45] are master regulators of bone formation. Runx2 induces OB differentiation and enhances their migration via activation of PI3K-AKT signaling [49]. Although genetic studies suggested a hierarchy between Runx2 and Osterix in bone formation, evidence also points to autonomous effects of each factor in regulating skeletal cell differentiation [50, 51]. In support of this concept, in the YF mice, our data showed differential expression of Runx2 and Osterix during reparative osteogenesis (Fig 5). The results further suggest that the enhanced bone formation in response to fracture may be due to increased expression of Osterix. However, the regulation of Osterix downstream of the Cbl-PI3K interaction appears to be independent of Runx2. Another recent study demonstrated that total loss of Cbl resulted in enhanced Osterix expression in osteoblastic cell lines [52]. This comes in support of our underlying hypothesis that Cbl may regulate bone formation through direct enhancement of Osterix expression. Additionally, this group found that Osterix can directly interact with Akt, and upon phosphorylation by the kinase, becomes more stable and translocates into the nucleus where it induced higher expression of its target genes [53]. This can at least partially explain the increased bone within the remodeling fracture callus of YF mice compared to WT mice. The mouse model we used in this study is a global knock in mutant in which only the interaction between Cbl and PI3K is abrogated. Therefore, we believe that the observed phenotype including the upregulation of Osterix in the periosteum of fractured mice is not likely to result from changes in Cbl’s expression level or its ubiquitin ligase function. The observed increase in Osterix expression in the YF mice is in agreement with published literature indicating a role for Cbl regulation of Osterix in bone via the PI3K signaling pathway [52]. Our observation of increased bone formation during fracture healing in the absence of Cbl-PI3K interaction is also supported by the recent finding that mice lacking PTEN in osteoblasts have improved intramembranous and late endochondral fracture healing, demonstrating that increased PI3K signaling in osteoblasts results in increased bone formation [54].

We initially reported significant increases in peak moment and yield displacement in intact bones from adult YF mice compared to wild type controls, suggesting that structural and geometrical changes in YF bones contribute to whole bone strength [30]. Torsion testing of intact femora in adult YF mice also exhibited an increase peak torque and work to failure while these parameters were significantly decreased in YF fractured femora in the initial stages of callus bone remodeling. The increased bone formation, and delayed remodeling observed over the course of fracture healing may explain the decreased strength of YF fractured femora. As remodeling progressed, the strength of WT and YF fractured femora resolved to no significant difference when the bones are fully healed. We speculate that the lack of Cbl-PI3K interaction results in a delay of restoration of strength in fractured femora due to improperly remodeled bone at earlier time points when bone remodeling is delayed.

The YF mutant callus had increased TRAP staining and significantly more osteoclasts during the remodeling phase of fracture healing. While this increase in osteoclast number was expected, expression of OC markers TRAP and Cathepsin K were also increased in YF fracture calluses despite the delay in remodeling the soft as well as the bony callus. These findings are consistent with our initial characterization of the skeletal phenotype of YF mice demonstrating that the lack of Cbl-PI3K interaction resulted in increased OC numbers with impaired resorptive capacity [27–29, 31]. PI3K signaling is known to regulate osteoclast bone resorption as osteoclast-specific deletion of the p85 genes results in an osteopetrotic phenotype caused by a defect in the bone-resorbing activity of osteoclasts [55]. The lack of Cbl-PI3K interaction in OCs leading to impaired resorption can also partially explain the increased callus size and increased bony callus volume observed in YF mice compared to WT and the delay in restoration of strength due to delayed remodeling of the bony callus. PI3K signaling is known to play an important role in skeletal formation and dynamic bone remodeling [11, 12].

Thus we discovered that upon fracture, YF mice have increased bony callus volume (**Figs 2 and 3**) as well as increased incorporation of fluorochrome labels into de-novo repaired bone on day 14 and 21 post–fracture (**Fig 5C**). Also, we found that the YF callus had decreased erosion surface/osteoclast surface, which is indicative of decreased resorption (Table 1). Together, the data indicate that the larger callus in YF mice is due to the combination of increased bone formation and delayed bone resorption.

Genetic studies showed that PI3K and its downstream target AKT are critical regulators of skeletal development [56, 57]. IKKα is also a downstream target of the PI3K signaling cascade [58], and its binding to NFκB, results in its retention in the cytosol and prohibiting it from repressing its transcriptional targets, including Osterix [59]. Additionally, small GTPases Rac1 and Rac2 have been shown to become activated downstream of PI3K signaling [48, 53]. Both GTPases have been implicated in osteoblast proliferation, differentiation, and apoptosis [60], and osteoblast migration [48]. We observed significant increases in expression of each of these downstream targets of PI3K signaling within cells of the YF fracture callus (Fig 7), suggesting that PI3K signaling is enhanced when Cbl binding to PI3K is abrogated. Furthermore, this enhanced signaling may be contributing to the OB and OC phenotypes observed during fracture healing in our current study, and explain the increased bone formation and delayed remodeling of the fracture callus.

To our knowledge, this fracture study is the first to describe a role for PI3K signaling in dynamic bone remodeling from a global context. Although the effect of enhanced PI3K signaling in OBs appears to enhance OB numbers and bone formation, it also decreases OC function despite increased numbers. The combination of these effects results in a delay in fracture healing. One cannot discount that age-related changes in bone remodeling may also be affected by altered PI3K signaling. Future investigations will also focus on abrogating bone resorption in the context of fracture healing via administration of anti-resorptive drugs to the YF mice to better dissect the cellular mechanisms involved in the observed osteogenic phenotype. Moreover, because the YF mutation is global, thereby affecting all cell types, it is difficult to define the cell autonomous effects that contribute to fracture healing. Future studies employing conditional YF mutants will enable us to assess cell autonomous effects of Cbl-mediated PI3K regulation on fracture healing.

## Supporting Information

S1 Fig Radiographic Scoring of Fractured Femora Indicates Enhanced Initial Response to Fracture in the Absence of Cbl-PI3K Interaction. Radiographic images of fractured femora at 7, 14, 21, and 28 days post-fracture were scored by two independent blinded reviewers for **A.** Periosteal and Endosteal Reaction **B.** Callus Opacity and **C.** Cortical Remodeling and Bridging. n>5 *p<0.05 vs. WT.
(TIFF)

S2 Fig 3D Reconstructions of MicroCT Scans of Fractured Femora Illustrate Increased Mineralization of the Callus in Mice Lacking Cbl-PI3K Interaction. 2D Scans of fractured femora from WT and YF mice at 14 and 21 days post-fracture were stacked, and 3D reconstructions generated to visualize mineralized tissue in the fracture callus. Representative image for each genotype is shown.
(TIFF)

S3 Fig Calcein Blue Stained Sections of Fracture Calluses Identify More Mineralized Tissue in YF Calluses at 14 days post-fracture. 5 micron frozen sections of fractured femora from WT and YF mice were stained with Calcein Blue to visualize mineralized tissue within the fracture callus.
(TIFF)

S4 Fig Cells Lacking Cbl-PI3K Interaction have larger TRAP^+^ cells. Sections (5 μM) of fractured femora from WT and YF mice were subjected to TRAP staining to identify osteoclasts within the fracture callus. 40x magnified images of representative osteoclasts (pink) in WT and YF calluses.
(TIFF)
