# Supplementary figures and images for: Role of Cbl-PI3K Interaction during Skeletal Remodeling in a Murine Model of Bone Repair
Source: PLoS One. 2015 Sep 22;10(9):e0138194. doi: 10.1371/journal.pone.0138194 (PMC4578922; doi:10.1371/journal.pone.0138194)

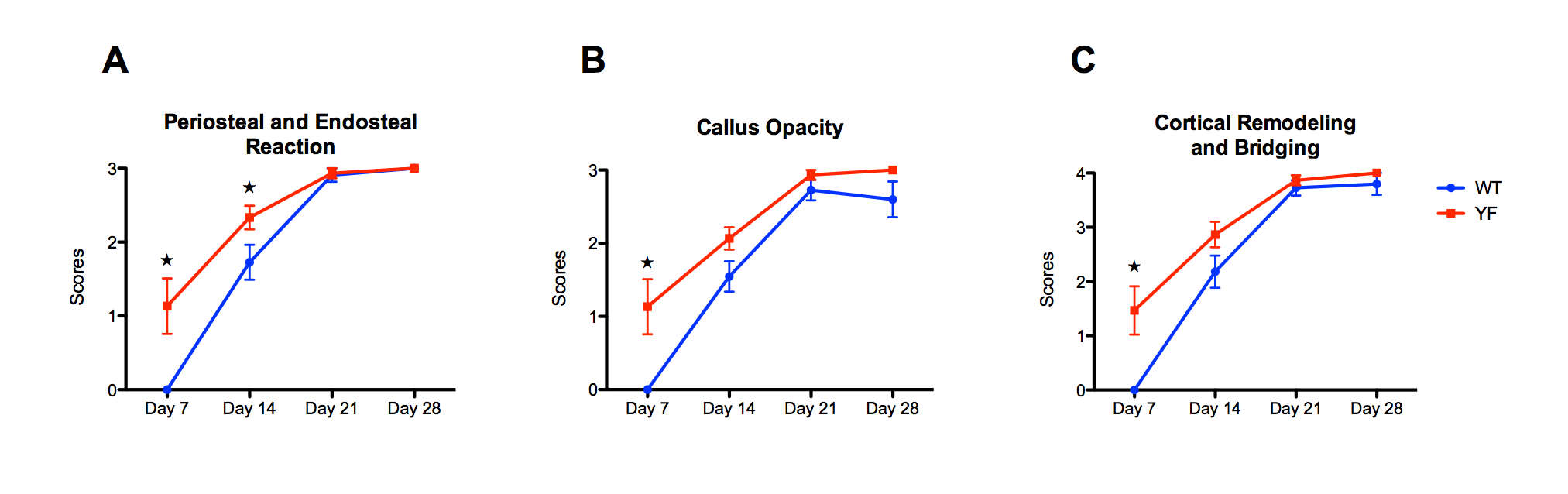

Supplement: S1 Fig — Radiographic images of fractured femora at 7, 14, 21, and 28 days post-fracture were scored by two independent blinded reviewers for A. Periosteal and Endosteal Reaction B. Callus Opacity and C. Cortical Remodeling and Bridging. n>5 *p<0.05 vs. WT. (TIFF) [file pone.0138194.s001.tiff]

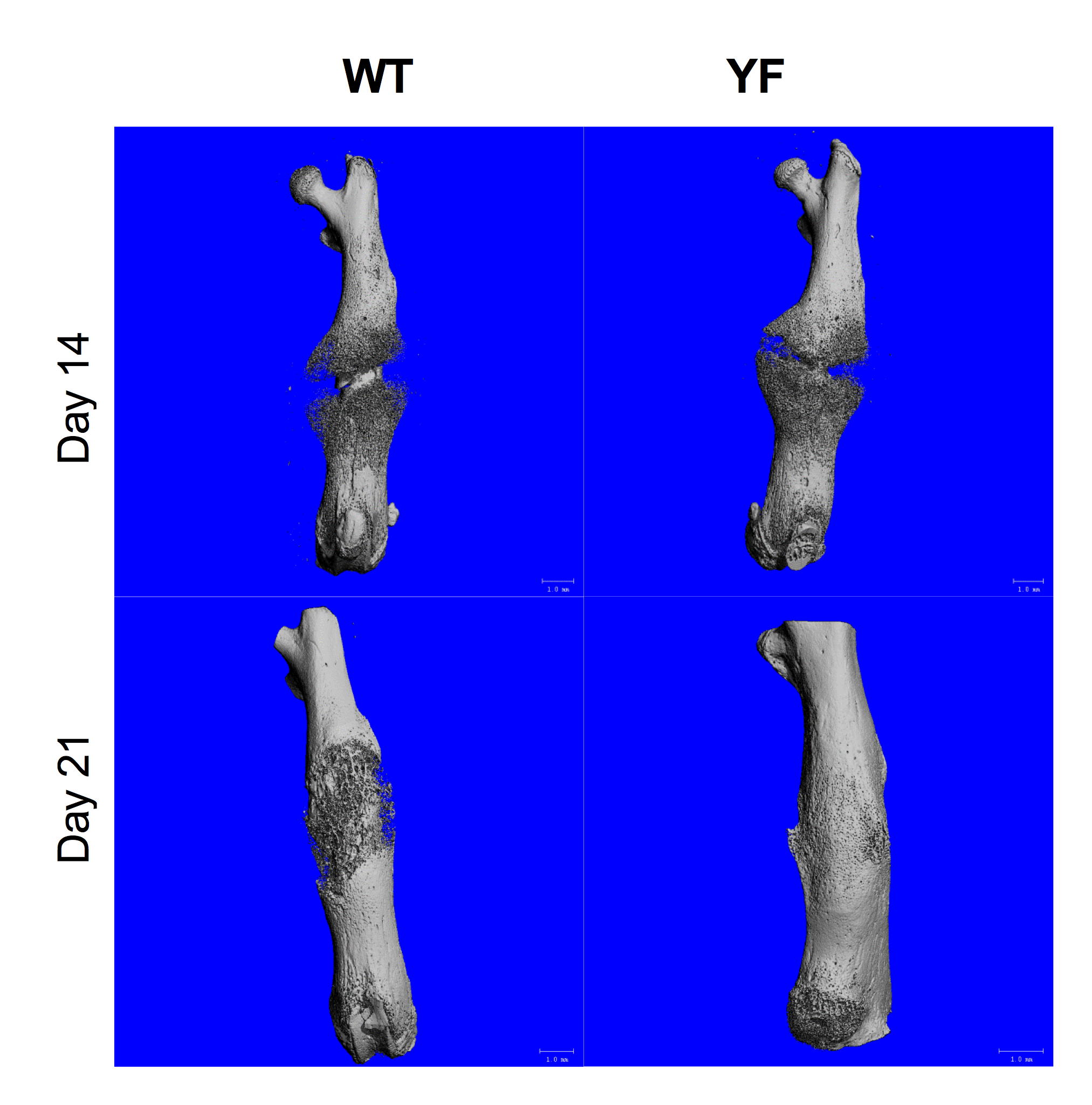

Supplement: S2 Fig — 2D Scans of fractured femora from WT and YF mice at 14 and 21 days post-fracture were stacked, and 3D reconstructions generated to visualize mineralized tissue in the fracture callus. Representative image for each genotype is shown. (TIFF) [file pone.0138194.s002.tiff]

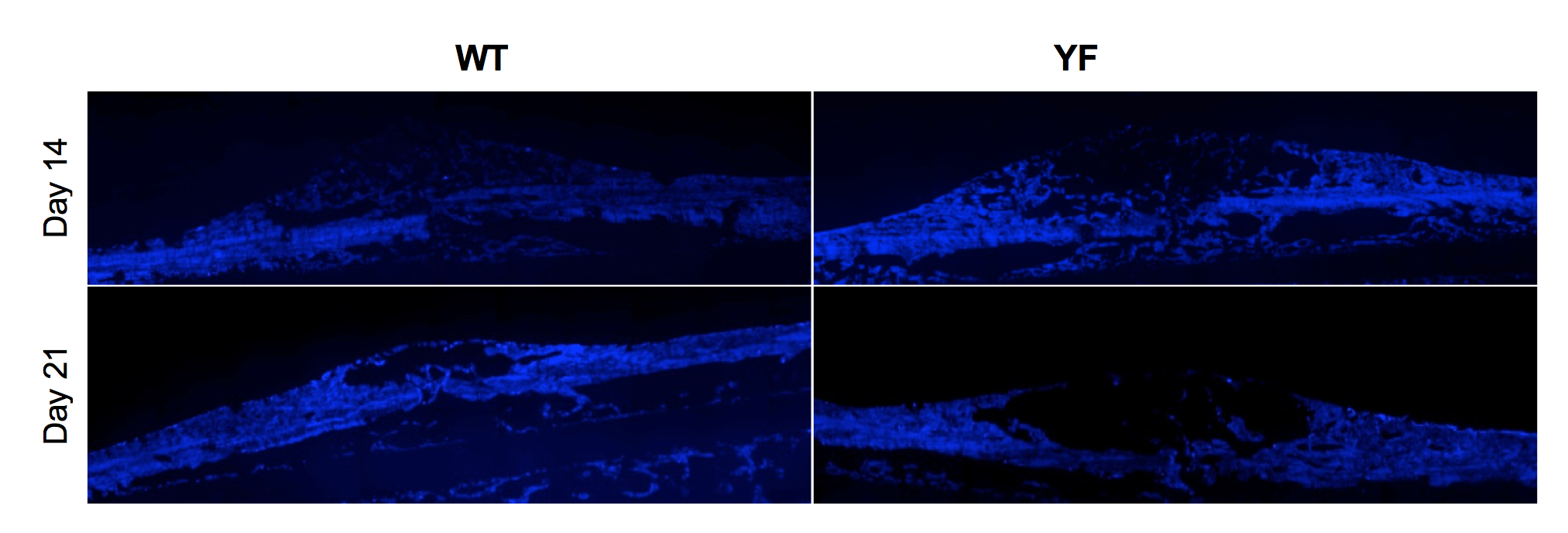

Supplement: S3 Fig — 5 micron frozen sections of fractured femora from WT and YF mice were stained with Calcein Blue to visualize mineralized tissue within the fracture callus. (TIFF) [file pone.0138194.s003.tiff]

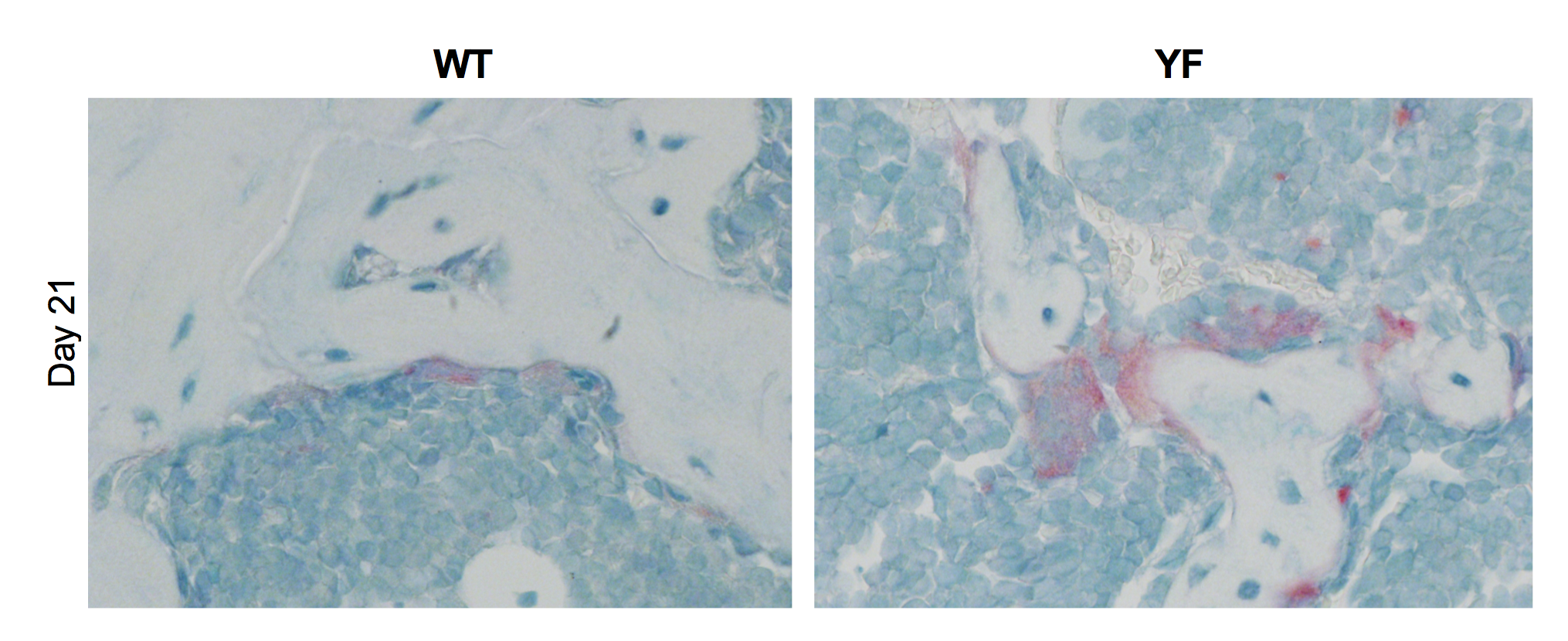

Supplement: S4 Fig — Sections (5 μM) of fractured femora from WT and YF mice were subjected to TRAP staining to identify osteoclasts within the fracture callus. 40x magnified images of representative osteoclasts (pink) in WT and YF calluses. (TIFF) [file pone.0138194.s004.tiff]
